# Non celiac gluten sensitivity and diagnostic challenges 

**Published:** 2018

**Authors:** Giovanni Casella, Vincenzo Villanacci, Camillo Di Bella, Gabrio Bassotti, Justine Bold, Kamran Rostami

**Affiliations:** 1 *General Practioner National Health Italy *; 2 *Institute of Pathology Spedali Civili Brescia Italy*; 3 *Pathology Department, Carate Brianza Hospital, ASST-Vimercate (Monza Brianza), Italy*; 4 *Gastroenterology and Hepatology Section, Department of Medicine, University of Perugia School of Medicine, Perugia, Italy*; 5 *Department of Gastroenterology Milton Keynes University Hospital, Milton Keynes, UK*; 6 *Allied Health and Social Sciences, University of Worcester, UK*

**Keywords:** Celiac disease, Non celiac gluten sensitivity, Wheat allergy

## Abstract

Non-celiac gluten sensitivity (NCGS), also referred to as non-celiac wheat sensitivity (NCWS), is a clinical syndrome characterized by both intestinal and extra-intestinal symptoms responsive to the withdrawal of gluten-containing food from the diet. The aim of this review is to summarize recent advances in research and provide a brief overview of the history of the condition for the benefit of professionals working in gastroenterology. Academic databases such as PubMed and Google Scholar were searched using key words such as ”non-celiac gluten sensitivity”, “gluten related disorders”, and the studies outlined in reference page were selected and analysed.

Most of the analysed studiers agree that NCGS would need to be diagnosed only after exclusion of celiac disease and wheat allergy, and that a reliable serological marker is not available presently. The mechanisms causing symptoms in NCGS after gluten ingestion are largely unknown, but recent advances have begun to offer novel insights. The estimated prevalence of NCGS, at present, varies between 0.6 and 6%. There is an overlap between irritable bowel syndrome and NCGS with regard to the similarity of gastrointestinal symptoms. The histologic characteristics of NCGS are still under investigation, ranging from normal histology to slight increase in the number of T lymphocytes in the superficial epithelium of villi. Positive response to gluten free diet for a limited period (e.g., 6 weeks), followed by the reappearance of symptoms after gluten challenge appears, at this moment, to be the best approach for confirming diagnosis. The Salerno expert criteria may help to diagnose NCGS accurately in particular for research purposes but it has limited applicability in clinical practice.

## Non Celiac Gluten Sensitivity 

Non-celiac gluten sensitivity (NCGS) is a clinical syndrome characterized by both intestinal and extra-intestinal symptoms responsive to the withdrawal of wheat and related cereals from the diet (1). These symptoms have been found to relapse following a gluten challenge NCGS may be diagnosed only after exclusion of celiac disease (CD) and wheat allergy as an established serological marker is not yet available. NCGS is often suspected by the patients themselves leading to self-diagnosis and self-treatment (2). It is important to consider that wheat, in addition to gliadin, contains a number of other potentially bioactive components that may cause gastrointestinal symptoms such as amylase trypsin inhibitors (ATIs) (3) and fermentable oligo-disaccharides-monosaccharides and polyols (FODMAPs) (4). A large number of IBS patients appear to respond to GFD, suggesting that gluten-containing food may be a trigger for IBS symptoms in at least a significant subset of patients (5). 

**History of NCGS**

The concept of NCGS was described for the first time in 1978 by Ellis* et al*. (6). These authors reported some patients with abdominal pain and diarrhea without histological duodenal lesions who improved after GFD. Similarly, in 1980, Cooper *et al*. (7) reported 8 females with abdominal pain, diarrhea and normal duodenal histology who again improved with GFD, with reoccurrence of symptoms following gluten challenge. The consensus findings of the first international expert meeting where NCGS has been defined was published in 2012 (8). 

**Composition of Wheat**

White Flour comprises about 80% starch and 10% protein (9). The indigestible oligosaccharides such as fructo-oligosaccharides and fructans constitute 13.4% of the dietary fiber in wheat (9) the latter contains also a considerable amount of indigestible ologisacharides galactans (9). Gluten represents 75-80% of the wheat proteins and it comprises 2 major groups: the glutenin and the gliadin proteins. Due to their amino acid sequences, the gluten proteins are partially resistant to digestion in the upper gastrointestinal tract, thus resulting in the formation of various peptides with a high degree of potential immunogenicity in the small intestine (10). 

**Pathophysiology of NCGS**

The mechanisms that determine symptoms in NCGS after gluten ingestion are still largely unknown, but it is evident that there are marked differences with CD, a condition that is related to an autoimmune process with adaptive immune system activation, as well as wheat allergy (WA), which is IgE-dependent (1). Sapone *et al*. (8) (6) suggests that intestinal permeability and adaptive immune system may have a less pronounced role in NCGS than in CD. Having said that, the presentation of NCGS is not yet fully understood and this issue is controversial as we explain below. 

It is worth noting that gluten has been found to have some intrinsic biologic properties (11) causing alteration of cellular morphology and motility (12), as well as cytoskeleton organization and intercellular contact through the tight junction proteins (13). 

Some Gliadin peptides bind the TLR2 receptor that increase Interleukin 1 production, a proinflammatory cytokine, through the mediation of Myd88 (14). MyD88 is a key protein mediating the release of zonulin in response to gluten ingestion and this increases the mucosal permeability. NCGS patients show higher levels of TLR2 compared to those with CD, causing dysbiosis similar to that observed in the pathogenesis of inflammatory bowel disease (IBD) (15). 

The intestinal cells exposed to gluten show a reduced survival, as suggested by increased apoptosis and a reduction of nucleic acid (DNA and RNA) and protein synthesis (16).

A study by Junker *et al*. (3) pointed the attention toward other molecules in wheat, the ATIs, capable of triggering the Toll-like receptor 4 pathways leading to the release of proinflammatory cytokines (3). 

In addition, non-protein components of wheat, the fermentable oligo-disaccharides-monosaccharides and polyols (FODMAPs), have been found to cause non-specific gastrointestinal symptoms in the context of NCGS (1). 

In 2016, Uhde, *et al*. (17) showed that individuals with wheat sensitivity display highly increased serum levels of intestinal fatty acid-binding protein, in conjunction with elevated levels of soluble CD14, lipopolysacchide (LPS)-binding protein, and antibodies to bacterial LPS and flagellin, all of which declined in response to the elimination of gluten-containing food (i.e., wheat, rye, and barley) from diet. The study provided clear evidence for an immunological mechanism underlying NCGS distinct from CD, whereby intestinal epithelial cell damage leads to microbial tranalsocation and extensive systemic immune activation. The study also provided a panel of objective serologic markers with diagnostic potential. Because circulating microbial LPS is known to bind directly to TLR4 on the luminal surface of brain blood vessels with increased local cytokine secretion (18), the findings may also explain some of the neuropsychiatric symptoms related to NCGS. 

**Genetic of NCGS**

 Up to half of NCGS patients may have genes coding for the HLA DQ2 and/or DQ8 molecules. HLA DQ2/DQ8 are present in 95% of all celiac patients and their absence can be used to rule out the diagnosis of CD in 95% of all cases (8). HLA DQ2-DQ8 are present in 30% of Healthy subjects. As such, it does not appear to be a significant association between the celiac disease HLA markers and NCGS.

**Clinical Diagnosis of NCGS**

Volta *et al* (19), in a multi-center Italian study of 486 patients responsive to a gluten-free diet (GFD), reported that most patients showed associated gastrointestinal and extra-intestinal symptoms. Bloating and abdominal Pain were the most important gastrointestinal symptoms (> 80%), whereas more than 50% of these patients reported diarrhea, 27% alternating bowel habits and 24% constipation (19). Other symptoms included epigastric pain, nausea, aerophagia, gastro-esophageal reflux disease and aphtous stomatitis. Tiredness, lack of well-being, neuropsychiatric symptoms as headache, anxiety, “foggy mind”, arm/leg numbness, depression, muscle or joint pain, weight loss, dermatitis, skin rash featured prominently among the extraintestinal symptoms of NCGS. There was an “overlap” between irritable bowel syndrome (IBS) and NCGS with regard to symptoms (20). In patients with clinical characteristics compatible with Rome III criteria for IBS, particularly in those with diarrhea, NCGS is diagnosed in a high percentage of cases; (21). Shahbazkhani *et al*. (22) found a large percentage of IBS patients (83%) to be gluten sensitive in their trial (72 patients). Also in patients with allergic disorders, a high prevalence of NCGS was reported by Massari *et al.* (77 NCGS/262 allergic patients) (23). The reported prevalence of NCGS, at this moment, varies between 0.6 and 6% (1). Anti-gliadinliadin antibodies may be present in about 50% of patients with suspected NCGS (24). Based on Salerno expert criteria, the patient may have 1 to 3 main symptoms that are quantitatively assessed using a Numerical Rating Scale with a score ranging from 1 to 10 (21, 25). The double blind placebo controlled gluten challenge (8 g/day) includes a one-week challenge followed by a one-week wash-out of strict GFD and a new crossover to the second one-week challenge (25). The vehicle for the challenge should contain cooked, homogeneously distributed gluten (25). 

A variation of 30% in 1 to 3 main symptoms between the gluten and placebo may discriminate positive from negative results (25). The Salerno Criteria (25) is the most reliable tool so far for diagnosing NCGS. However, it has a limited applicability in clinical practice outside the clinical trials. Most of the clinicians use an open gluten challenge fashion to ascertain the diagnosis of NCGS. An open gluten challenge is the most practical way forward, despite reduced diagnostic accuracy. Further study would be needed to introduce a pragmatic and more practical policy to help clinician to diagnose NCGS in absence of more sensitive and specific biomarkers.

**Figure 1.**
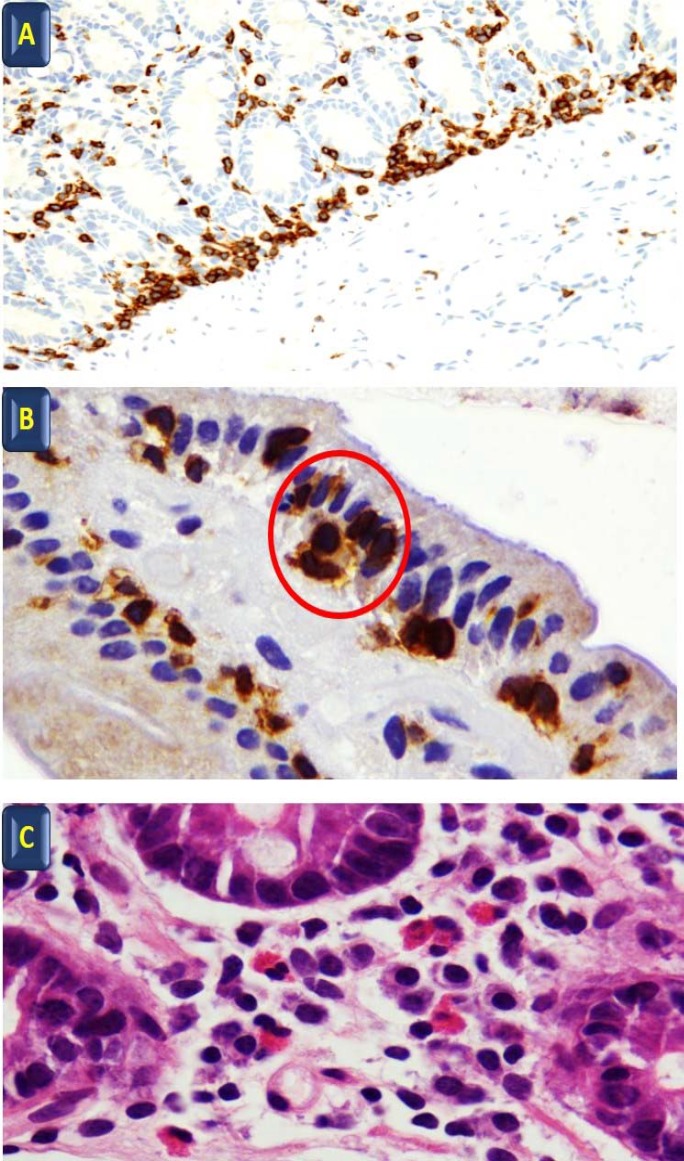
A: Linear disposition of T lymphocytes at the base of the mucosa CD3 immunohistochenistry 40x; B: Cluster of ¾ lymphocytes in the superficial epithelium (red circle) CD3 immunohistochemistry 100x; C: Eosinophils in lamina propria H&E 100 x

The clinical observation in Salerno expert criteria includes an administration of a modified version of the Gastrointestinal Symptom Rating Scale (GSRS) based on reviews of gastrointestinal symptoms and clinical symptoms almost largely used to evaluate common symptoms of Gastrointestinal Disorders (26). The patient identifies 1 to 3 main symptoms that may be evaluated using a Numerical Rating Scale (NRS) with a score ranging from 1 (mild) to 10 (severe). The instruction includes item evaluating also extra intestinal symptoms. Elli *et al*. (27) performed a multicenter double-blind-placebo controlled trial with crossover enrolling 134 patients (17 males and 117 females); 98 of these patients underwent a gluten challenge after 3 weeks long GFD and 28 of these, all females, reported a symptomatic relapse and deterioration of quality of life.

## Duodenal Histology of NCGS

The histologic characteristics of NCGS are still under investigation, ranging from reports of apparently normal histology to slight increase of T lymphocytes in the superficial epithelium of normal villi (28). In a recent editorial, Talley and colleagues (29) reported the presence of high intraepithelial lymphocytes (IEL) and increased duodenal eosinophils in some cases of NCGS, but underlined the overlap of these findings in patients with functional gastrointestinal disorders (29). Villanacci V *et al.* (30) have reported that patients with NCGS might have a normal number of T lymphocytes but a peculiar disposition of this cells in small “cluster” of ¾ elements in the superficial epithelium and a linear disposition in the deeper part of the mucosa together with an increased number of eosinophils in lamina propria (Villanacci V unpublished data). A slight increase of gamma delta T cell receptors in NCGS has also been reported by some studies (1). Brottveit *et al.* (31) studied the initial mucosal immunologic events in CD and NCGS patients before and after a gluten challenge, showed an increase of interferon gamma and Heat Shock Protein 27 levels after gluten challenge and confirmed the presence of a higher density of intraepithelial lymphocytes. Bucci *et al.* (32) did not found any gliadin related immunologic alteration in the duodenal mucosa of NCGS patients. In NCGS patients, basophil activation was reported to be positive in 66% of patients responding to wheat blind challenge and to be associated with duodenal intraepithelial lymphocytosis and eosinophilic infiltration of the duodenum and the colon (33). Sub-microscopic changes or duodenal Intraepithelial Lymphocytosis may be present in about 50% of NCGS cases under definition of Microscopic Enteritis (34). 

**Table 1:** Gluten free diet effect assessed by double-blind placebo-controlled oral gluten challenge trials in IBS patients (authors and reference number first column

Authors	No of IBS	Study design	Am Gluten	Outcome
Cooper et al. 1980 (7)	6	DBPC	(20 g/d)	Sig Improv
Biesiekierski et al. 2011 (41)	34	DBPC	(16 g/d)	Sig Improv
Carroccio et al. 2012 (34)	276	DBPC	(13 g/d)	Sig Improv
Shahbazkhani et al (22)	72	DBPC	52g/day	Sig Improv
Di Sabatino et al. 2015 (38)	61	DBPC	(4.375 g/d)	Sig Improv
Zanini et al. 2015 (37)	35	DBPC	(10g/d)	Sig Improv
Elli et al. 2016 (27)	98	DBPC	(5.6 g/d)	Sig Improv
Barmeyer et al 2016 (40)	35	Observational		90%
Zanwar et al (39)	60	DBPC		

Abbreviations: Sig Improvement: significant improvement, DBPC: double blind placebo controlled; IBS: irritable bowel syndrome

**Figure 2:**
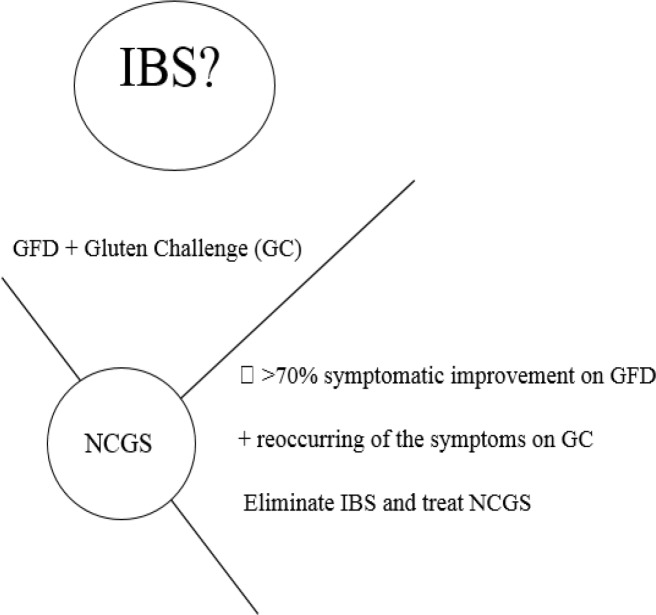
Studies listed in table 1 suggest over 70% of patients with irritable bowel syndrome (IBS) respond to gluten free diet (GFD) with reoccurring the symptoms on gluten challenge (GC). For targeted and effective treatment it is essential to recognise and differentiate non-coeliac gluten sensitivity from IBS

## Open Questions

The lack of standardized biomarkers remains an important challenge in the diagnosis of NCGS. The clinical symptoms are not specific and they may be confused with other conditions like IBS (1). The current algorithm recommends the exclusion of CD and wheat allergy and symptomatic improvement on GFD (25). 

Principal remaining questions pertain to what is triggering NCGS and why some people suddenly become gluten intolerant. The factors implicated in the occurrence of NCGS remain largely unknown and it is unclear who is susceptible to this condition. It has been suggested that dysbiosis following a gastroenteritis might count as a risk factor for NCGS (35). 

The future research agenda should explore the genetic background, histological characteristic, susceptibility and risk factors for NCGS in addition to developing reliable biomarkers. It is essential to separate NCGS from IBS as IBS is a non-specific condition and IBS therapies not only are not effective in NCGS, but also these medications and their side effect may impair the quality of life of NCGS patients and adversely drain the healthcare resources (36-42). See table 1 and figures 1, 2.

## Conflict of interests

The authors declare that they have no conflict of interest.
